# Unraveling the Link of Altered TGFβ Signaling with Scoliotic Vertebral Malformations in Osteogenesis Imperfecta: A Comprehensive Review

**DOI:** 10.3390/jcm13123484

**Published:** 2024-06-14

**Authors:** Angelos Kaspiris, Elias S. Vasiliadis, Georgios Tsalimas, Dimitra Melissaridou, Ioanna Lianou, Fotios Panagopoulos, Galateia Katzouraki, Michail Vavourakis, Ioannis Kolovos, Olga D. Savvidou, Evangelia Papadimitriou, Spiros G. Pneumaticos

**Affiliations:** 1 Third Department of Orthopaedic Surgery, School of Medicine, National and Kapodistrian University of Athens, “KAT” General Hospital, Nikis 2, 14561 Athens, Greece; eliasvasiliadis@yahoo.gr (E.S.V.); georgetsalimas@yahoo.com (G.T.); gkatzouraki@hotmail.com (G.K.); michail.vavourakis@outlook.com (M.V.); kolovioan@gmail.com (I.K.); spirospneumaticos@gmail.com (S.G.P.); 2 Laboratory of Molecular Pharmacology, Group for Orthopaedic Research, School of Health Sciences, University of Patras, 26504 Patras, Greece; epapad@upatras.gr; 3 First Department of Orthopaedic Surgery, School of Medicine, National and Kapodistrian University of Athens, “ATTIKON” University Hospital, Rimini 1, 12462 Athens, Greece; dimitramelissaridi@gmail.com (D.M.); olgasavvidou@gmail.com (O.D.S.); 4 Department of Orthopaedic Surgery, “Rion” University Hospital and Medical School, School of Health Sciences, University of Patras, 26504 Patras, Greece; jolianou@hotmail.com (I.L.); panfo97@gmail.com (F.P.)

**Keywords:** Osteogenesis Imperfecta, scoliosis, bone metabolism defects, bone mineral density, Transforming Growth factor-β

## Abstract

Osteogenesis Imperfecta (OI) is a genetic disorder caused by mutations in genes responsible for collagen synthesis or polypeptides involved in the formation of collagen fibers. Its predominant skeletal complication is scoliosis, impacting 25 to 80% of OI patients. Vertebral deformities of the scoliotic curves in OI include a variety of malformations such as codfish, wedged-shaped vertebrae or platyspondyly, craniocervical junction abnormalities, and lumbosacral spondylolysis and spondylolisthesis. Although the precise pathophysiology of these spinal deformities remains unclear, anomalies in bone metabolism have been implicated in the progression of scoliotic curves. Bone Mineral Density (BMD) measurements have demonstrated a significant reduction in the Z-score, indicating osteoporosis and a correlation with the advancement of scoliosis. Factors such as increased mechanical strains, joint hypermobility, lower leg length discrepancy, pelvic obliquity, spinal ligament hypermobility, or vertebrae microfractures may also contribute to the severity of scoliosis. Histological vertebral analysis has confirmed that changes in trabecular microarchitecture, associated with inadequate bone turnover, indicate generalized bone metabolic defects in OI. At the molecular level, the upregulation of Transforming Growth factor-β (TGFβ) signaling in OI can lead to disturbed bone turnover and changes in muscle mass and strength. Understanding the relationship between spinal clinical features and molecular pathways could unveil TGFβ -related molecular targets, paving the way for novel therapeutic approaches in OI.

## 1. Introduction

Osteogenesis Imperfecta (OI), also termed “brittle bone disease”, is a genetically and clinically heterogenous connective tissue disorder that is accompanied by several types of skeletal dysplasia, growth retardation, decreased bone mass, osseous fragility, vertebral deformities, and recurrent fractures. OI occurs with a prevalence of approximately 1 in 10,000 subjects [1,2]. The extraskeletal clinical symptoms of OI include blue sclerae, dentinogenesis imperfecta, ligamentous laxity, muscle weakness, hearing loss, reduced ventilatory capacity, vascular fragility, and heart valve diseases [1]. Most cases of OI are caused by mutations in the COL1A1 and COL1A2 genes that encode procollagen type I protein [3], but mutations of non-collagen genes also contribute to OI pathogenesis, although they are detected in under 10% of affected cases [4]. The classification of OI patients with primary collagenous defects was based on Sillence criteria, which consider the following aspects: (a) the type of inheritance, (b) radiographic manifestations, and (c) the clinical characteristics of the affected individuals. Specifically, Type I OI encompasses patients with autosomal dominant inheritance and blue sclerae, while Type II individuals have perinatal lethality, crumpled femora, and rib beading. Types III and IV are characterized by progressive skeletal deformities and normal sclerae, respectively [5]. Mutations in the IFITM5 gene, also called BRIL (bone-restricted IFITM-like protein), are responsible for the development of an autosomal dominant type V of OI disease [6]. The IFITM5 gene encodes an osteoblastic-specific protein that has a pivotal role in osteoblastic maturation, matrix mineralization, and bone formation [7]. The distinct clinical phenotype of patients with OI type V is characterized by the formation of a hyperplastic bone callus during fracture healing, calcification of the interosseous membrane of the forearm, irregularly arranged lamellae, and mesh-like histology [7]. During the last decade, progress in genetic sequencing techniques and bioinformatics technologies led to the discovery of additional dysregulations of non-COL1A1/2 genes in OI subjects that either affected the post-translational modification (PTM) and package of collagenous fibers, such as mutations in cartilage-associated protein (CRTAP) or prolyl-3-hydroxylase-1 (P3H1) or Bone Morphogenetic Protein 1(BMP1) genes [8], or osteoblastic differentiation, such as secreted protein acidic and rich in cysteine (SPARC), transcription factor SP7, and WNT1 [9], and the mineralization of the collagenous matrix, such as Interferon-induced transmembrane protein 5 (IFITM5) and Plastin-3 (PLS3) [10].

An important clinical finding of OI is scoliosis. The incidence of scoliotic deformities in OI individuals ranges from 25 to 80% [1,11,12,13,14,15,16]. Van Dijk and Sillence [17] reported that scoliotic curve progression was correlated with severe types of OI, while Liu et al. noted that age, reduced Bone Mineral Density (BMD), Bone Mineral Content (BMC), trabecular number, increased mechanical strains, and joint hypermobility may affect scoliotic severity [4]. Lower leg discrepancy, pelvic obliquity, hypermobility of the spinal ligaments, or microfractures of the vertebrae may also result in spinal malformations in these subjects. Moreover, the severity and progression of scoliotic curves in OI patients are associated with the genotypes and mutations, as well as the age and Bone Mineral Density (BMD) of the affected individuals [18]. Although the underlying mechanisms of scoliotic development in OI remain unclear, many researchers have examined the implication of several signaling pathways and molecular factors, including growth factors such as insulin-like growth factors (IGF-1), transforming growth factor beta (TGFβ), fibroblast growth factors (FGFs), platelet-derived growth factor (PDGF), or cytokines such as interleukins (ILs) and tumor necrosis factor (TNF). TGFβ is an important osseous-derived factor belonging to the TGFβ superfamily that also includes activins, inhibins, and bone morphogenetic proteins (BMPs). TGF-β is implicated in bone remodeling as it couples the bone formation and resorption processes and recruits osteoprogenitor cells to the site of resorption [19,20,21]. Mutations that affect the structure or functionality of the molecules involved in TGFβ signaling are often associated with the pathogenesis of scoliosis [22,23,24,25], while defects in TGFβ signaling pathways can lead to several connective tissue disorders, including Marfan, Ehlers-Danlos, Loeys-Dietz and Shprintzen–Goldberg syndromes [26], that severely affect the musculoskeletal system, accompanied by severe scoliotic deformities [26]. Specifically, the above-mentioned heritable connective tissue disorders originated from loss-of-function mutations in fibrillins that constitute significant structures of the extracellular matrix and regulate a wide variety of processes taking place during skeletal development, immune responses, and osseous tissue homeostasis, accompanied by increased TGFβ activation [26].

Several studies also reported impaired TGFβ signaling in OI [19,27], suggesting this as a possible target for treatment interventions [19]. Therefore, it could be crucial to highlight the potential correlation of a defective TGFβ pathway with scoliosis pathogenesis. Given the scarcity of the literature reports investigating the association between scoliotic progression and bone metabolic impairment in OI, the purpose of our review study was to provide data examining: (a) the clinical presentation and associated demographic data, (b) the characteristic biochemical bone metabolic alterations associated with the TGFβ pathway, and (c) possible cellular and molecular targets that can modify the scoliotic progression in OI patients.

## 2. Clinical and Bone Metabolic Characteristics

Although spinal deformities are a common finding in OI patients, the incidence and severity of scoliosis depend on the type of OI [4]. Patients with Type III OI present an increased frequency and severity of spinal malformations when compared to individuals suffering from Types I and IV [1,4,28,29,30,31,32,33]. The study by Patel et al. [28] involving 544 patients with OI reported that subjects suffering from Type III exhibited a higher prevalence of dentinogenesis imperfecta, severe scoliosis, and long bone deformities when compared to those with OI types I and IV. The study by Chen et al. reported that patients with the COLI1A1 and COL1A2 genotypes were linked to milder forms of scoliosis, while in patients with IFITM5 mutations, the above pattern was not displayed [18]. Interestingly, among the two collagen groups, COL1A2 was associated with decreased progression rate to severe scoliotic curves than COL1A1 [18]. Similarly, the association between joint hypermobility and scoliosis in severe types of OI was also confirmed by the cross-sectional one-center study by Apronen et al. [1], indicating that spinal joint hypermobility may contribute to the progression of scoliosis in OI. These results were also confirmed by a study by Ben Amor et al. [15] that revealed mild scoliotic changes in the presence of a mild OI phenotype.

**Figure 1:**
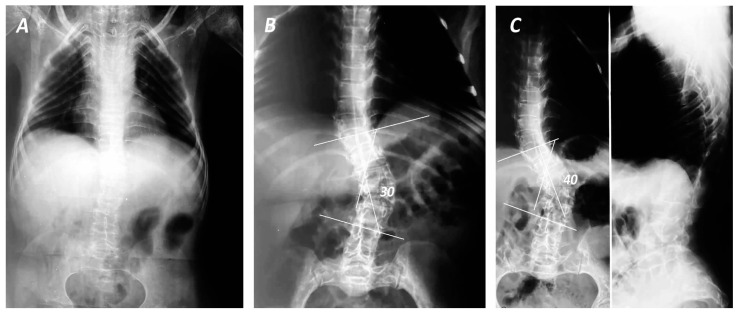
Anteroposterior (**A**–**C**) and lateral (**C**) views of total spine in standing position of a female patient with OI after 30 years of follow-up. Although during childhood the patient presented mild scoliosis (**A**), the deformity progressed rapidly during the last 5 years of follow-up (**B**,**C**), demonstrating right thoracolumbar curves (Lenke V) of 30 (**B**) and 40 degrees between 11th thoracic (T11) and 4th lumbar (L4) with apex in 2nd Lumbar (L2) vertebrae (**C**).

Although the age of onset of spinal deformities varies in international literature, ranging from 2 to 65 years [4], it is documented that curve progression is observed after the 5th year of age (Figure 1), and it worsens when the Cobb angle exceeds 50 degrees. This notion was corroborated by the study of Norimatsu et al. [34], which identified rapid progression of spinal deformities in patients with the congenital type of OI after the age of five, reaching peak severity at approximately 12 years of age. In the same study, scoliotic deformities in patients with the tarda type of OI developed gradually; however, a rapid deterioration was observed once the curve exceeded 50 degrees, with the curvature potentially escalating to more than 100 degrees [34]. The anatomical location and severity of the scoliotic curvatures were also associated with disease severity, as a cross-sectional study of 102 OI patients demonstrated that the prevalence of scoliosis was 74.5%, with a mild curvature of <40 degrees in 56 patients, moderate of <60 degrees, severe of <80 degrees and a very severe deformity of >80 degrees in 8, 7 and 5 patients, respectively [16]. The above study detected that the average expansion concerned 6.7 vertebrae (3–12), accompanied by an average rotation of 2 (1–4) [16]. These results were in line with the study of Watanabe et al. [32], which reported an average Cobb angle of 25.2 degrees (ranging from 5 to 108 degrees).

Scoliosis in OI is correlated with vertebral malformations, such as compression fractures, codfish or wedged-shaped vertebrae or platyspondyly [16], craniocervical junction abnormalities, and lumbosacral spondylolysis and spondylolisthesis [35,36,37,38]. Moreover, the analysis of lateral radiographic views demonstrated four types of vertebral body malformations in the OI spine, including biconcave, flattened, wedged, and unclassifiable vertebrae [39]. Interestingly, the presence of six or more biconcave vertebrae before puberty may result in severe scoliotic deformities (more than 50 degrees) [39].

Many recent studies have focused on the link between altered bone metabolism in OI and scoliotic deformities. An inverse correlation between the extent of scoliotic curvatures and the Z-score BMD as well as the thoracic kyphosis angle has been detected [35]. Engelbert et al. also observed a remarkably lower Z-score BMD in OI patients [30], but a correlation between osteopenia and scoliotic deformities was not established. Additionally, treatment with intravenous pamidronate in children with OI prevented the development of severe scoliosis, while the presence of low BMD Z-scores at the L2–4 levels or vertebral deformities at the coronal and sagittal level and the increased percentage of corrective osteotomies at the lower extremities were linked to the development of severe scoliosis [40]. Similarly, Palomo et al. examined the long-term outcomes after bisphosphonate administration (pamidronate or zoledronic acid) for at least 6 years in OI patients, demonstrating the association between increased Z-scores and BMD in the lumbar spine and the frequency of vertebral reshaping [41]. These are also confirmed by the study by Sato et al., which showed a reduced scoliosis progression rate in OI Type III after bisphosphonate therapy but no positive effect on scoliosis in OI Types I and IV [42]. Compressed vertebral reshaping was more prominent when intravenous bisphosphonates were administered to growing patients, whereas in adults, no significant beneficial effect was observed [43]. Pamidronate treatment also prevented further vertebrae compression fractures, maintaining the spinal damage in children with all OI types, irrespective of age or collagen I mutation type [44]. In animal models of OI and Ehlers-Danlos syndrome mice (Col1a1^(Jrt)/+^ mice), reduced BMD and BMC were not only strongly associated with scoliotic deformities compared with wild-type mice but also resulted in early and progressive anomalies of vertebral body morphology [45]. Although a direct etiological relationship between the severity of vertebral compression and scoliotic curvatures in OI was not established [14], vertebral osseous fragility or collapse and the concurrent damage to vertebral endplates [46] may partially explain spinal deformities in these individuals [47].

## 3. TGFβ Signaling in OI

Although Scoliosis is considered a multifactorial spinal deformity, several studies have reported the close association between low bone mineral density, altered vertebral growth, dysfunctional paraspinal muscle activity, and the development or progression of scoliotic curves. Additionally, vertebral osteopenia is indicated as a prognostic factor of scoliotic severity [48]. Much evidence supports that the Transforming growth factor β (TGFβ) signaling is implicated either in the regulation of osteoblastic function or in muscular activity, playing a key role in the maintenance of bone mass volume and neuromuscular activity.

**Figure 2:**
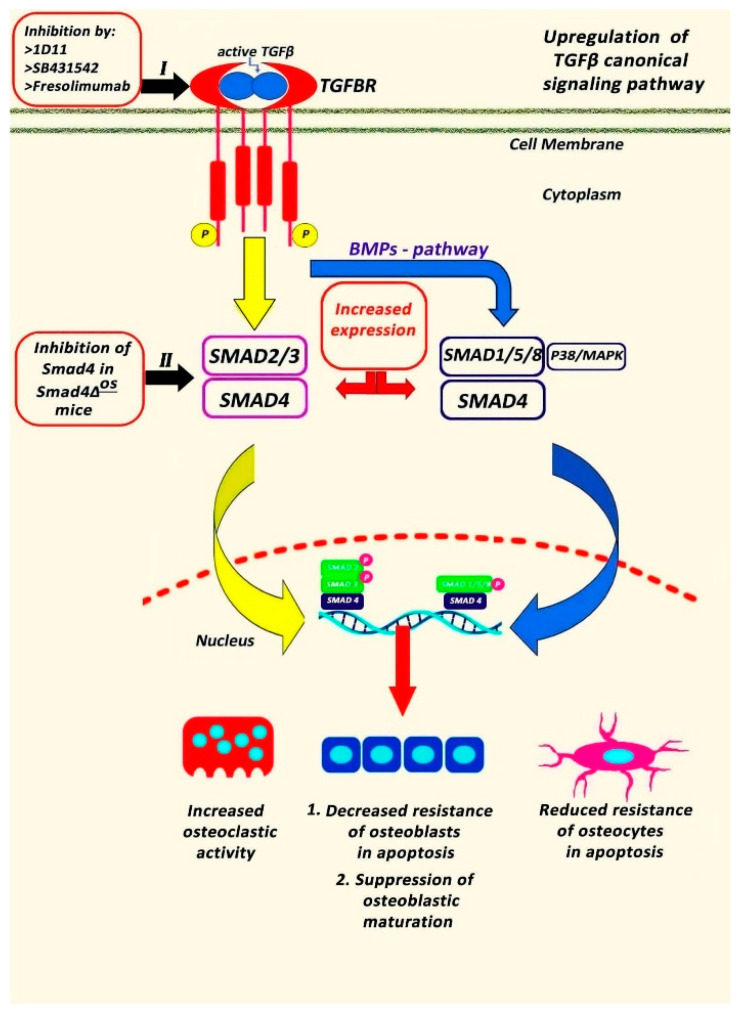
The Transforming Growth Factor beta (TGFβ) pathway in Osteogenesis Imperfecta (OI). Upregulation of TGFβ signaling is the characteristic finding in OI, resulting in increased SMAD 2 and 4 phosphorylation. SMAD4 phosphorylation leads to increased osteoclastic activity and osteoblastic and osteocyte apoptosis as well as in suppressed osteoblastic maturation [21]. Inhibition of TGFβ signaling pathway at TGFβ receptor with Fresolimumab [21], 1D11 [27] or SB431542 [66] repressed the SMAD inhibitory signaling and induced the BMP signaling resulting in increased production of alkaline phosphatase and decreased number of osteoclasts [27]. Similarly, Smad4-knock out mice showed significant increase in osteoblast and osteocyte number [63]. TGFβ, Transforming Growth Factor beta; TGFR, Transforming Growth Factor beta Receptor; BMP, Bone Morphogenetic Proteins; BMPR, Bone Morphogenetic Proteins Receptor; MAPK, mitogen-activated protein kinase; TF, Transcriptional Factor.

Transforming growth factor β (TGFβ) is expressed in the skeletal system, playing a critical role in bone development and osseous homeostasis [26]. Abnormal TGFβ signaling caused by mutations in the TGFβ genes was linked to the development of several genetic disorders characterized by skeletal malformations [26], revealing its significant involvement in bone remodeling and integrity. Although TGFβ1 is mainly expressed in human tissue [48], it must be clarified that TGFβ is secreted in three isoforms (1, -2, and -3), displaying a distinct binding affinity for TGFβ receptors and serving specific functions in vivo [26]. Initially, TGFβ ligands are secreted as precursor polypeptides but then go through proteolytical cleavage [49]. TGFβ synthesis is followed by the formation of disulfide dimers, which are subsequently linked to latency-associated peptide (LAP) [50]. Afterwards, the LAP-TGFβ complex binds to the latent- TGFβ-binding protein (LTBP) developing the TGFβ large latent complex (LLC) [50]. When LLC is released to the extracellular space, it interacts with fibrillin I through LAP, not only regulating the expression and functionality of TGFβ but also maintaining the structural integrity of connective tissues [51]. Thus, mutations of fibrillin I can modify TGFβ activity and lead to TGFβ signaling-related connective tissue disorders [26]. Proteolytic cleavage, low pH, or interactions with reactive oxygen species and integrins result in the disconnection of TGFβ from LAP, generating mature TGFβ that can bind with TGFβ receptors, initiating downstream signaling [52]. The primary receptors through which TGFβ exerts its effects are TGFβR1 and TGFβR2, both of which exhibit similar structures [50]. These receptors, characterized as serine/threonine kinase receptors, can form heteromeric complexes and interact with various TGFβ isoforms [53]. Binding of TGFβ to TGFβR triggers either the canonical TGFβ signaling cascade that is mediated by intracellular SMAD proteins [54] or the non-canonical pathway that includes mitogen-activated protein kinase (MAPK) pathways, such as extracellular signal-regulated kinases (ERK), c-Jun N-terminal kinase (JNK), and p38 MAPK [55] (Figure 2).

Defective TGFβ signaling pathways have been documented in OI mouse models, supporting the hypothesis that the upregulation of TGFβ signaling could play a central role in the pathogenesis of OI, affecting osteoblastic and osteoclastic activity. Specifically, in vivo experiments in Crtap(−/−) mice revealed increased expression of TGFβ target genes and a higher ratio of phosphorylated Smad2 to total Smad2 protein and Smad2 reporter activity in the skeleton. Furthermore, the same study found that the binding ability of type I collagen to the small leucine-rich proteoglycan decorin, a known regulator of TGFβ activity, was decreased [56]. Similarly, a study with type III OI patients observed increased expression of TGFβ [21]. The immunohistological examination of bony tissue also detected increased immunostaining of phosphor -SMAD2 due to the upregulation of TGFβ signaling in OI type III patients [18]. Moreover, administration of anti- TGFβ antibodies, such as 1D11, in jck mice models with renal osteodystrophy was associated with a decreased pSMAD2/SMAD ratio, TGFβ signaling and bone turnover in the femur [57]. Increased expression of TGFβ and SMAD3 phosphorylation also induced the number of osteoprogenitors cells and reduced the number of differentiated osteoblasts, resulting in low bone matrix quality in vivo [58]. TGFβ can also be connected to proteoglycan peptides and to collagen fibrils, indicating a close relationship between TGFβ and collagen integrity [59,60]. The notion that the increased expression of TGFβ signaling was correlated with wide dysregulation of the osteocyte transcriptome in *Crtap*^−/−^ and oim/oim [61] as well as *in Brtl^+/−^* mice models, which have a heterozygous point mutation in *Col1a1* [62], led researchers to apply the 1D11 anti- TGFβ antibody to OI in vivo [27]. Inhibition of TGFβ in dominant OI mice models carrying a *col1a2* gene mutation at G610C resulted in increased trabecular bone volume fraction and suppression of serum biomarkers of osteoblast differentiation, osteocalcin, Type I Collagen Cross-Linked C-Telopeptide (CTX-1) and bone formation indices [27]. The same study also detected a remarkable reduction in the immunostaining of Tartrate-resistant acid phosphatase (TRACP) and the number of osteoclasts per bone surface area, suggesting that TGFβ pathway inhibition repaired the high-turnover process and improved biomechanical properties in OI mice [27]. Similarly, in SmadΔ^os^ mice, Col1α1- Cre—mediated disruption of Smad4 resulted in reduced osteoblastic and osteocytic apoptosis and reduced osteoclastic function [63]. Currently, the safety and effectiveness of fresolimumab, an anti-TGFβ antibody (SAR439459), are under clinical trial investigation in adults with OI Types I and IV.

Two other members of the TGFβ superfamily that interact with ECM molecules are myostatin and activin A, which suppress muscular growth and strength [63,64]. Moreover, a study by Goh et al. [65], examining skeletal phenotypes in activin receptor (ACVR) type 2A knock-out mice, showed increased trabecular bone, suggesting a direct and negative regulation of activin signaling on bone mass, too. Indeed, in +/oim mice deficient in myostatin (+/mstn +/oim), myostatin deficiency resulted in increased body weight, muscular mass and improved bone microarchitecture and biomechanical properties compared to +/oim mice [62]. Based on the understanding that muscle weakness and reduced bone strength are primary clinical features of individuals with OI, targeting the activin signal pathway might offer an alternative therapeutic approach for both muscles and bones in OI. This is also supported by the notion that overexpression of TGFβ signaling in patients with fibrillin deficiency such as Marfan’s syndrome, which is characterized by severe paraspinal muscular myopathy and atrophy, is accompanied by severe forms of scoliotic curvatures [48]. It must be highlighted that more than 60% of individuals with Marfan’s syndrome develop scoliosis [48].

Crosstalk between TGFβ and BMP signaling pathways has also been reported (Figure 2), suggesting that inhibition of the endogenous TGFβ pathway was accompanied by enhanced BMP expression after the administration of the TGFβ type I receptor kinase inhibitor SB431542 to C2C12 mouse cells [66]. Although the exact role of the BMP signaling cascade remains unclear, the above TGFβ inhibitor suppressed the expression of inhibitory Smad-6 and-7 and upregulated the BMP-4 and-6 mRNA levels during the maturation phase of osteoblastic differentiation, leading to increased production of alkaline phosphatase, bone sialoprotein, and matrix mineralization [66,67]. The significance of BMP signaling is also supported by the findings that the intraosseous application of BMP-2 in the tibial diaphysis promoted periosteal formation in *Col1a2^+/G610C^* mice [68]. This is further corroborated by a recent study indicating that the administration of BMP-2 to OI mice was associated with considerable increases in bone volume. However, it was not effective in promoting fracture healing or enhancing the strength of the callus neocortex [69]. Interestingly, a recently discovered mutation identified in association with OI involves the *tent5A* gene, which encodes the FAM46A protein [70]. This protein is a member of the nucleotidyltransferase (NTase) fold superfamily and functions as a BMP modulator. It has been demonstrated to promote BMP signaling through the stabilization of the SMAD1/SMAD4 complex, consequently inhibiting neural crest formation [71,72]. However, the exact crosstalk pathways between TGFβ and BMP signaling in OI need further investigation.

The TGFβ signaling pathway has been found to be significantly elevated in OI human and experimental animal myoskeletal tissues, suggesting its central role in OI pathogenesis. Given that TGFβ alters not only the bone microenvironment but also the muscular and ligamentous tissues, it is plausible to suggest that it may contribute to the development of vertebral deformities and scoliosis progression in OI patients.

## 4. Conclusions

Collectively, the upregulation of TGFβ and the impairment of osseous turnover led to a progressive decrease in bone mass and generalized bone metabolism defects in OI patients. Recent experimental and clinical studies have revealed an increased incidence of osteopenia or osteoporosis in OI individuals with scoliosis, resulting in severe vertebral malformations and compression fractures. Although genetic or epigenetic factors may affect the presence of spinal dysplasia, bone metabolic impairment may contribute to the severity of deformities in the anatomic locations where increased mechanical forces are applied, such as the spine or tibia. Given that scoliotic curves were correlated with dysplastic vertebrae phenotype and defective vertebrae remodeling processes, TGFβ upregulation could be a significant contributing factor to the onset and progression of the scoliotic deformities in OI.

## Data Availability

The datasets analyzed during the current study are available from the corresponding author upon reasonable request.
